# Residue Levels of Pesticides on Fruits for Use in Wildlife Risk Assessments

**DOI:** 10.1002/ieam.4345

**Published:** 2020-10-21

**Authors:** Jens Schabacker, Joerg Hahne, Jan‐Dieter Ludwigs, Martin Vallon, Manousos Foudoulakis, Roger Murfitt, Kai Ristau

**Affiliations:** ^1^ RIFCON GmbH Hirschberg Germany; ^2^ Bayer AG, Crop Science Division Monheim Germany; ^3^ Corteva Agriscience, Agriculture Division of DowDuPont Chalandri Greece; ^4^ Syngenta, Jealott’s Hill International Research Centre Bracknell Berkshire United Kingdom; ^5^ BASF SE, Agricultural Solutions Limburgerhof Germany

**Keywords:** Pesticides residues, RUD, Fruits, Environmental risk assessment, Birds, Mammals

## Abstract

The guidance document on risk assessment for birds and mammals (GD) provides generic residue values of pesticides on potential diet items for use in wildlife risk assessments. For most diet items, these values are based on a large number of residue studies. However, the default residues per unit dose (RUD; standardized for application of 1 kg substance per hectare) values for fruits were taken from a few literature trials of unclear relevance for regulatory purposes. These trials were conducted according to neither current European Union (EU) agricultural practice nor to recommendations given in the GD about how to conduct wildlife‐relevant residue studies. Therefore, field study data on fruit residue levels from applications of pesticides in fruiting crops were compiled and evaluated. Corresponding studies had been conducted during the last 26 y in the EU. In the final data set, 291 studies provided 1002 residue values in different fruits, including grapes, berries (currants, raspberries, gooseberries), fruits from orchards (apple, peach, pear, lemon, mandarin, orange, apricot, cherry, plum), gourds (pumpkins, cucumbers, squash, melons), and strawberries. This data set provides a basis for revising registration‐relevant RUD values for fruits as potential diet items for birds and mammals in environmental wildlife risk assessments. The objective of this study was to estimate the resulting residue levels in different fruits determined under field conditions following the application of pesticides across their growing areas within the EU in diverse climatic areas that can be used directly in wildlife risk assessments. The large data set of usually about more than 100 residue values per “fruit group,” all evaluated at EU member state level, revealed significantly lower RUDs compared to the current default RUDs presented in the GD. These new RUD values for fruits should be considered for use as default values in future bird and mammal risk assessments and in respective guidance documents. *Integr Environ Assess Manag* 2021;17:552–561. © 2020 The Authors. *Integrated Environmental Assessment and Management* published by Wiley Periodicals LLC on behalf of Society of Environmental Toxicology   Chemistry (SETAC)

## KEY POINTS

- Extensive data sets on pesticide residues in fruit are analyzed, which considerably increases the knowledge base in this area.
- The extent of the data sets used to calculate new fruit residues per unit dose (RUD) values is a major strength.
- This work provides valuable information for regulatory risk assessments and tests some of the underlying assumptions that play a role in the risk assessment of pesticides to birds and mammals.
- It adds to the ongoing revision of the European Food Safety Authority's guidance document on risk assessment for birds and mammals.

## INTRODUCTION

Potential negative effects of pesticides on nontarget wildlife need to be assessed according to the European Union (EU) Regulation 1107/2009 (EC 2009). For this purpose, the current European Food Safety Authority (EFSA) guidance document (GD) on risk assessment for birds and mammals (EFSA 2009) provides the general procedures to assess the risks to birds and mammals. The GD is intended to be revised in the near future (Lahr et al. 2018; EFSA 2019).

The current scheme of the European environmental risk assessment follows a tiered approach, starting with worst‐case assumptions. For a risk assessment on birds and mammals exposed to pesticides, a toxicological endpoint, determined in toxicological studies, is compared to exposure estimates for representative wild bird and mammal species (for more details, see Ludwigs et al. 2013; Brooks et al. 2017). The species‐specific exposure estimates result from model calculations in which the food intake (i.e., diet items and amount per fraction) and the concentration of pesticide residues in these diet fractions are considered.

Therefore, a key data set for the risk assessment given in the GD (Table 1 in Appendix F of EFSA 2009) are the presented default initial residue levels (expressed as residue per unit dose [RUD]) of pesticides on potential diet items after application. The RUD is defined as the residue concentration on the respective diet items as a result of a pesticide application, standardized for a rate of 1 kg substance per hectare.

**Table 1:** EFSA (2009) risk assessment scenarios for frugivorous birds and mammals

Crop group	Scenario	Generic focal species
Bush and cane fruit	Fruit stage BBCH 71–79	Frugivorous bird (blackcap)
Bush and cane fruit	Fruit stage BBCH 71–79	Frugivorous mammal (dormouse)
Fruiting vegetables	Fruit stage BBCH 71–89	Frugivorous bird (crow)
Fruiting vegetables	Fruit stage BBCH 71–89	Frugivorous bird (starling)
Fruiting vegetables	Fruit stage BBCH 71–89	Frugivorous mammal (rat)
Orchards	Fruit stage BBCH 71–79	Frugivorous mammal (dormouse)
Strawberries	Flowering/development of fruit/Maturity of fruit BBCH 61–89	Frugivorous bird (starling)
Vineyard	Ripening	Frugivorous bird (thrush/starling)

BBCH = Biologische Bundesanstalt, Bundessortenamt und CHemische Industrie scale; EFSA = European Food Safety Authority.

Although the default RUD values for green plant matter given in the GD are based on a relatively large number of registration‐relevant residue decline studies provided by industry during the compilation of the GD, the respective RUD values for other bird and mammal diet items are based on a rather limited database. This is particularly the case for fruits, where the current default values given in the GD are derived from only a small data set of unclear quality, published in Baril et al. (2005).

The actual intention of Baril et al. (2005) was to update earlier databases on residues, to examine whether extrapolating residue values across application rates is justified, and to establish a categorization of crop groups using crop morphology and cultivation methods. The authors established the following categories in fruiting crops: 1) small fruits from orchards (such as apricot, cherry, date, fig, kiwi, or plum), 2) large fruits from orchards (such as apple, lemon, mandarin, nectarine, orange, pear, or peach), 3) berries (such as black currant, blueberry, grape, or raspberry), 4) tomatoes, and 5) gourds. Strawberries (an important crop and potential fruit diet for vertebrates) were not covered.

However, the fruit residue data given in Baril et al. (2005) have several significant shortcomings. For instance, data were derived from studies with unknown design conducted in different regions of the world. Although more than 700 publications from the open literature were consulted for examination, only 25 field studies submitted by manufacturers to support registration were included in the database. Therefore, relevance for European regulatory purposes is strongly limited, given that most studies may not have been conducted in line with current EU agricultural standards or the GD (EFSA 2009; EC 2017). Further, the publication gives only a few details of the studies that contributed to the residue levels in the individual crop groups. For example, there is a lack of information on the Biologische Bundesanstalt, Bundessortenamt und CHemische Industrie (BBCH) growth stages (Meier 2018), which play an important role in today's risk assessment when it comes to classifying the various scenarios and selection of exposed (focal) species. Furthermore, with the exception of tomatoes, the derived RUD values for fruits in Baril et al. (2005) are based only on relatively few residue trials (e.g., *n* = 9 for berries, *n* = 33 for orchard fruits). Moreover, for some crops, such as strawberries, no crop‐specific residue data are available in Baril et al. (2005) and hence the corresponding default RUD value for strawberries in the GD is extrapolated from residue values for berries and grapes.

Hence, we consider it important to update and extend the residue data for fruits in order to have a robust data set available. We examined a large number of industry field studies conducted within the last 26 y on fruit residues after applications of modern pesticides in different fruit crops. These studies, actually conducted to define maximum residue doses for the metabolism and residues section of registration dossiers, provided initial residues recorded after application, which were used for the present evaluation. This large and comprehensive data set on fruit residues was derived from trials conducted in accordance with standard agricultural practice in diverse climatic areas across the EU during respective fruiting seasons. The initial (or maximal) residue values were then used as the basis for statistical analysis to derive representative RUD values in fruits as potential diet items for frugivorous birds and mammals.

In addition, we investigated in detail the relationship between the application rates used in these studies and the resulting residue levels. The RUD concept in the EFSA GD is based on a linear extrapolation of expected environmental concentrations across different application rates. Therefore, we tested whether this basic assumption is also fulfilled in the fruit residue data set here. Furthermore, we examined for each fruit category (according to the GD) whether the fruit species within each group show significant differences from each other.

Finally, we provide new default RUD values for environmental risk assessments for frugivorous scenarios of birds and mammals, which are considered more appropriate and reliable than the current default values (see Table 1 in Appendix F of EFSA 2009).

## METHODS AND STATISTICS

### Choice of relevant data

In total, 291 field studies (providing 1002 residue trials) conducted between 1991 and 2017 were available, measuring residue levels on fruits in different numbers of separate field trials (*n* = 1–8) per study after pesticide application (insecticides and fungicides) (Table 2). All study protocols followed regulatory‐relevant study guidance documents (e.g., OCSPP 860.1500 [OCSPP 2009], OECD TG 509 [OECD 2009]). Samples were collected on the day of applications and the following days. All studies were conducted for registration purposes, and only studies fulfilling the following criteria were included in the data set and considered for further analysis:

- Samples for residue analysis were taken at appropriate fruit ripening stages (see Table 1) on the day of application and shortly thereafter.
- Study was conducted under good laboratory practice (GLP) and evaluated at EU member state level as acceptable.
- Percentage recovery of spiked samples during analysis was not below 80%, ensuring that the residues found do not significantly underestimate the actual value.
- For “grapes” and “large fruits from orchards,” only trials with 1 application were considered due to the large number of available trials covering these crop groups.
- For “berries,” “gourds,” “small fruits from orchards,” and “strawberries,” trials with up to 8 applications were used. As a conservative approach, the first residue measurement directly after the last application was considered.

Relevant information regarding the pesticide used (active substance [a.s.]), application method, application rate, concentration of a.s., fruit type, BBCH growth stage (according to Meier et al. 2018), country, time of sampling, and the residue concentrations was extracted from each study report. Residue levels after last treatment (DALT0; i.e., on the day of application or shortly thereafter if maximum value was not measured at DALT0) were taken for further analysis.

The final data set consisted of 1002 initial or maximum residue values (each from 1 crop field trial conducted to determine the magnitude of the pesticide residue in fruits) from the following fruit types: grapes, currants, raspberries, gooseberries, apples, peaches, pears, lemons, mandarins, oranges, apricots, cherries, plums, pumpkins, cucumbers, squash, melons, and strawberries. The active substances used in 34% of the trials were insecticides and in 66% were fungicides.

The GD (EFSA 2009) defines specific frugivorous scenarios (5 for birds and 3 for mammals), grouping fruits with similar growing patterns, and therefore it is assumed that the exposure of species utilizing these fruit crop groups will be comparable (Table 1). The available residue data were therefore grouped into the existing GD‐relevant crop groups, defining the different diet items needed for calculating the exposure in the risk assessment. In general, for the frugivorous scenarios according to the GD, residue data from growth stages of development of fruits (BBCH 71–79) and maturity of fruits (BBCH 81–89) are required. For details on growth stages according to extended BBCH scales, see Meier (2018). Hence, from the available studies, those trials were selected that meet the requirements regarding the growth stages that have to be covered in the specific risk assessment scenarios (for an overview, see Table 3).

**Table 2:** Data set from field studies providing data on initial and maximum fruit residues after application of pesticides

EFSA (2009) GD crop group	Vineyard	Bush and cane fruit	Orchard		Fruiting vegetables	Strawberries	
Fruit group according to EFSA (2009) GD	Grapes	Berries^a^	Large fruits^b^	Small fruits^c^	Gourds^d^	Strawberries	Total
Nr of studies (*n*)	22	65	43	38	88	35	291
Nr of different active substances	10	9	10	10	12	6	n.a.^e^
Insecticides/fungicides (%)	43/57	28/72	99/1	34/66	10/90	31/69	34/66
Nr of trials (*n*)							
C‐EU	45	124	27	115	58	82	451
S‐EU	53	56	100	77	209	56	551
Total	98	180	127	192	267	138	1002
Maximum number of treatments before first sample	1	5	1	2	8	2	8

C‐EU = central European zone; EFSA = European Food Safety Authority; GD = guidance document; S‐EU = southern European zone.

^a^ Currants, raspberries, and gooseberries.

^b^ Apple, peach, pear, lemon, mandarin, and orange.

^c^ Apricot, cherry, and plum.

^d^ Pumpkins, cucumbers, squash, and melons.

^e^ Not applicable because the same substance can be used in different crops.

**Table 3:** New RUD values calculated for fruit groups according to EFSA (2009)

EFSA (2009) crop group	Vineyard	Bush and cane fruit	Orchard	Orchard	Fruiting vegetables	Strawberries
Fruit group analyzed	Grapes	Berries^a^	Large fruits^b^	Small fruits^c^	Gourds^d^	Strawberries
BBCH stages covered by evaluated studies	79–95	75–89	74–87	77–88	71–89	73–89
Nr of trials = residue values (*n*)	98	180	127	44	209	138
Mean RUD (SD)	1.6 (1.1)	5.0 (3.6)	0.9 (0.6)	2.8 (1.4)	0.7 (0.7)	1.2 (0.7)
Lower 95% confidence limit	1.4	4.4	0.7	2.4	0.6	1.0
Upper 95% confidence limit	1.8	5.5	1.0	3.2	0.8	1.2
Maximum	5.5	25.2	4.8	6.4	6.3	3.8
90^th^ percentile	2.9	9.2	1.5	4.3	1.3	2.2
Median	1.3	4.5	0.7	2.6	0.6	1.0
Minimum	0.2	0.4	0.2	0.8	0.0	0.1

BBCH = Biologische Bundesanstalt, Bundessortenamt und CHemische Industrie scale; C‐EU = central European zone; EFSA = European Food Safety Authority; RUD = residue per unit dose; S‐EU = southern European zone.

^a^ Currants, raspberries, and gooseberries.

^b^ Apple, peach, pear, lemon, mandarin, and orange.

^c^ RUD value from cherries (C‐EU), covering apricot and plum (C‐EU), and cherry, apricot, and plum (S‐EU); total of 192 trials.

^d^ RUD value from pumpkins, cucumbers, squash, and melons from studies conducted in S‐EU (covering 58 additional RUD values from C‐EU).

### Analysis of data

Data analysis was performed using Microsoft Excel (Microsoft Corporation 2010) and GraphPad InStat for Windows version 3.10 (GraphPad 2009).

The main principle in the analysis of the data was to obtain a coherent data set that is as large as possible (also for subsets) without overlooking potentially relevant differences between subsets of the data. Therefore, the data were examined with regard to identifying differences in residue levels attributable to recognizable fruit characteristics or groups of fruit within the groups of crops treated together in risk assessments under the current GD (EFSA 2009). However, differences between different substances (pesticides) are not investigated in detail because this distinction is not made in the GD (EFSA 2009).

We calculated the RUD for each residue value by dividing the highest measured value by the applied rate of pesticide (or the rate at last treatment in cases of more than 1 application) to be most conservative. Successive applications are usually undertaken to maintain an efficiency level. In any case, higher residues and therefore RUDs will result from multiple applications irrespective of application intervals and rates applied before. Hence, using the last application rate before first samplings will lead to a conservative estimation of the RUD.

Then we tested whether the data distributions of the resulting RUD values followed Gaussian distributions using the Kolmogorov and Smirnov method (Bortz et al. 1990). Because measured residues, as well as the calculated RUD value distributions, did not follow a normal distribution, we applied nonparametric tests: Mann‐Whitney Test (MWU) for comparing 2 unpaired groups and Kruskal‐Wallis Test (KW) to compare 3 or more unpaired groups (followed by Dunn's post‐test) on the residue data. All tests were conducted as 2‐tailed, and values of *P* < 0.05 were considered to indicate statistically significant differences. If tests did not indicate significance, the data were not separated but were further analyzed together in order not to split the data set into unnecessarily small groups.

We computed medians and quantiles as representative parameters per subset due to data distributions. However, means and standard deviation were also calculated and are given in table format as is done for the current default residue data in the GD (EFSA 2009). It should be noted that the GD (EFSA 2009) proposes mean (for chronic risk) and 90^th^ percentile RUD values (for acute risk) per diet fraction or fruit group, even if from a statistical point of view the median would be more representative for such data distributions, that is, if the data did not follow a normal distribution.

The data set was analyzed in terms of identifiable groups (subsets) within the regulatory‐relevant fruit groups to ascertain possible different residue loads due to the geographical area from which the data originate.

With regard to the EU pesticide risk assessment scheme in ecotoxicology, the EU is divided into 3 regulatory zones, North, Central, and South (EC 2017). Besides potential differences between fruit groups, we thus also paid particular attention to potential differences existing between residues from different regulatory zones within the same fruit group. All available trials were from either southern EU (S‐EU; 551 trials in the final data set) or central EU (C‐EU; 451 trials). According to the GD (EFSA 2009), France is considered part of the southern regulatory zone. However, in terms of residue data, France is split into northern and southern parts according to the European Commission (EC 2017) and European and Mediterranean Plant Protection Organization climatic zones (EPPO 2014) to better reflect the differences in Mediterranean and Atlantic maritime conditions within the country. In line with this approach, we assigned all trials conducted in northern France (N‐FR) to the C‐EU zone and all trials conducted in southern France (S‐FR) to the S‐EU zone subset of data.

Within the regulatory‐relevant groups of berries, gourds, and fruits from orchards, we examined whether the appearance and surface texture of fruits potentially had any impact on residue levels.

Because for berries, gourds, small fruits from orchards, and strawberries, trials with up to 8 applications were used, testing was done to check whether the number of applications has a significant effect on the level of residues, which would mean that pooling the data is not appropriate.

When calculating the exposure, the RUD values are multiplied by the respective application rate according to the application pattern. Therefore, all subsets or fruit groups, respectively, were examined to determine whether the basic assumption of a linear relationship between application rate and resulting residue levels was confirmed. This was done because a linear relationship makes it possible to use the residue data derived from any study at any application rate. Hence, a linear function should at least approximately describe the relationship with sufficient reliability. To test for linearity, we performed a linear regression and then tested whether the data differ significantly from the determined linear equation with the runs test (RT) (Wald and Wolfowitz 1940) determining the number of series of consecutive points whose residuals are either all positive or all negative (runs) related to the regression line.

In cases where there was no direct linearity in the relationship between application rate and residues, we checked whether the calculated exposure under the assumption of linearity (RUD concept of the GD, taking into account the number of applications and a corresponding application interval) overestimated or underestimated the measured values. Therefore, the calculated residues were compared with the measured residues at the same application rate. To be conservative in terms of risk assessment, it was therefore requested that a higher proportion (at least >2/3) needed to exhibit the higher values than those measured by calculating the residues according to the RUD method.

## RESULTS

The purpose of the present analysis was to investigate the relationship between pesticide application rates and the residue levels in fruits that are treated together in risk assessments as determined in crop groups. Therefore, the results are presented with regard to the crop groups as given in the GD (EFSA 2009).

### Grapes: EFSA (2009) crop group “vineyards”

For grapes, as crop group “vineyards” according to the GD (EFSA 2009), a data set of 98 residue values from fruit samples collected in a comparable stage of development (BBCH 79–95) was available. As for all other crop groups according to the GD EFSA (2009), 2 subsets of data can be assumed from a regulatory perspective based on the different regulatory zones: S‐EU (with residue trials available here from southern France, Italy, Spain, Portugal, Greece) and C‐EU (trials from Germany, northern France). However, the results from S‐EU and C‐EU showed nearly identical residue medians (1.4 mg/kg S‐EU; 1.3 mg/kg C‐EU), which did not differ significantly from each other (MWU, *P* = 0.17). In addition, no significant deviations from linearity were found for both S‐EU (RT, *P* = 0.47) and C‐EU (RT, *P* = 0.44). This confirms that both data sets meet the requirements for RUD calculation according to the GD (EFSA 2009). Therefore, it was considered appropriate to combine the data of both regulatory zones and derive an overall RUD value for grapes, covering all cultivation areas (see Table 3).

### Fruits from orchards: EFSA (2009) crop group “orchards”

Temperate orchards consist of perennial tree plantings that include pome fruits and stone fruits (Extended BBCH scale, Meier 2018). Crop trees that fall into the GD (EFSA 2009) crop group “orchards” are diverse in fruit types and characteristics such as size and morphology. Particularly the volume–surface relations and details of the structure of the skin may potentially influence the residue level. Large fruits from pome fruit and citrus orchards, such as apple, peach, pear, lemon, mandarin, and orange, normally exceed 5 cm in diameter and are consumed by frugivorous and omnivorous small animals in small pieces only. Stone fruits such as cherries are smaller and can be swallowed as a whole, at least by some species. In the GD (EFSA 2009), different residue values are given for small and large fruits from orchards. Also in our data set there is a significant difference between residues of apple, peach, pear, lemon, mandarin, and orange (i.e., large fruits), and apricots, cherries, and plums (i.e., small fruit; MWU, *p* < 0.0001). Therefore, we followed the classification in the GD (EFSA 2009) according to fruit size for further analysis.

#### Large fruits from orchards

In the category of large fruits from orchards, 127 residue values from apple, peach, pear, lemon, mandarin, and orange have been combined. The developmental stages of the fruits analyzed ranged from BBCH 74 to 87 (including growth stages of maturity of fruits), thus only slightly exceeding the BBCH range given for the corresponding frugivorous scenario in orchards according to the GD (EFSA 2009), which is BBCH 71 to 79 (until the final stage of fruit development). Large fruits from orchards exhibit a variety of fruit types and morphologies. However, no differences between RUDs of individual fruit samples (such as apples and pears with a rather plain and even surface, and peaches with a hairy and fuzzy surface) were found (Dunn's test; *P* > 0.05). Therefore, these potential subsets were grouped. The runs test indicated no significant deviation from linearity (RT, *P* = 0.10), confirming that the residue data in large orchard fruit meet the requirements for the RUD calculation according to the GD (EFSA 2009). The medians of residue data from S‐EU (for this fruit group, France, Italy, Spain, Portugal, and Greece) and C‐EU (Germany, northern France) did not differ significantly (MWU, *P* = 0.62). It was therefore considered acceptable to combine the zonal data to derive an overall RUD value for large fruits from orchards (see Table 3).

#### Small fruits from orchards

In the GD (EFSA 2009) residue category of “small fruits from orchards,” fruits such as apricot, cherry, and plum have been combined (*n* = 192). The developmental stages of the fruits analyzed range from BBCH 77 to 88, thereby slightly exceeding the fruit growth stages in the GD crop group “orchards” (i.e., BBCH 71–79). Measured residue levels of studies with 1 or 2 applications, based on sampling taken on the day of application, did not differ significantly from each other (MWU, *P* = 0.129). The runs test again indicated no significant deviation from linearity (RT, *P* = 0.08), confirming that the residue data in small orchard fruit meet the requirements for the RUD calculation (see EFSA 2009).

The S‐EU data were available from southern France, Italy, Spain, and Greece and C‐EU data from Germany, northern France, Austria, the Netherlands, Belgium, Hungary, Poland, the Czech Republic, and the United Kingdom for small fruits from orchards. Here, the residue levels of the 2 geographical subsets based on the different regulatory zones differed significantly from each other (MWU, *P* < 0.02), with a median of 2.0 mg/kg for S‐EU (*n* = 77) and a median of 1.4 mg/kg for C‐EU (*n* = 115). In the S‐EU, apricots accounted for about 67% of the residues, whereas in the C‐EU, cherries and plums accounted for 84% of the residues. Therefore, the data for further analysis were kept separately by location.

Comparing differences between RUDs of individual fruit samples (such as apricots, cherries, and plums), variation among different fruits was apparent (KW, *P* < 0.001). Plums exhibited significantly lower residue levels (median 0.6 mg/kg, *n* = 52, C‐EU; and 0.4 mg/kg, *n* = 13, S‐EU) than either cherries (median 2.8 mg/kg, *n* = 44, C‐EU; and 1.1 mg/kg, *n* = 13, S‐EU, MWU) or apricots (median 2.3 mg/kg, *n* = 19, C‐EU; and 2.2 mg/kg, *n* = 51, S‐EU, MWU) (Dunn's test; *P* < 0.01).

Therefore, it seems justified to give separate values for plums and cherries and apricots as a combined group, respectively. Nevertheless, for pragmatic reasons, a single value is proposed (the highest among the different groups; see Table 3) as a conservative approach that covers all group‐specific RUD values in small fruits.

### Gourds: EFSA (2009) crop group “fruiting vegetables”

Fruits such as pumpkins, cucumbers, squashes, and melons are combined in the EFSA GD as gourds. The (final) application took place during fruit development and maturity of fruits and seeds (BBCH 71–89), thus matching the requirements for fruiting vegetables scenarios as given in the GD (EFSA 2009). With 267 residue values, this data set was the largest. No difference in residue levels between round and spherical fruits such as squashes and melons or elongated fruits such as zucchinis or cucumbers was found (MWU, *P* = 0.85). The number of applications (up to 8 treatments in some studies) also had no significant influence on the residue level measured on fruit samples after the last application in each case (KW, *P* = 0.91). Therefore, the runs test and the other analyses were done with the full data set, irrespective of fruit shape or number of applications.

Runs test did not indicate significant deviation from linearity (RT, *P* = 0.46). This confirms that residues in gourds meet the requirements for the RUD calculation. The comparison of the 2 geographical subsets revealed significant differences in residue data between studies conducted in S‐EU (data from southern France, Italy, Spain, Portugal, and Greece) with a median of 0.7 mg/kg (*n* = 209) and C‐EU (from Germany, Belgium, Hungary, Poland, Switzerland, and northern France) with a median of 0.4 mg/kg (*n* = 58; MWU, *P* < 0.05). In the interests of a conservative approach and for pragmatic reasoning, the data set leading to the higher of the 2 values is selected to provide a single value for risk assessment for gourds (see Table 3).

### Berries: EFSA (2009) crop group “bush and cane fruit”

In the EFSA (2009) GD category “berries,” different fruits such as currants, raspberries, and gooseberries have been combined. In all the studies we analyzed, the applications were made during fruit development and ripening (BBCH 75–89) and the data thus meet the requirements of the respective risk assessment scenarios of BBCH 71 to 79 for frugivorous birds and BBCH 71 to 89 for frugivorous mammals. The data set with 180 residue values is considered heterogeneous in relation to fruit types, sizes, and details of the skin structure. However, no significant difference was found between, for example, currants and raspberries (MWU, *P* = 0.74), indicating that size and skin structure had no significant influence on the residue content. For gooseberries only a limited number of residue measurements were available (*n* = 3), which excludes any meaningful statistical comparison.

The number of applications (up to 5) did not significantly influence the final RUD values (KW, *P* = 0.098). No deviations from a linear model were indicated for S‐EU (RT, *P* = 0.78) and C‐EU (RT, *P* = 0.062). This confirms that both data sets meet the requirements for the RUD calculation. The median RUDs of S‐EU (from southern France, Spain, Italy) and C‐EU (from Belgium, Germany, the United Kingdom, Poland, the Netherlands, Hungary, and northern France) did not differ significantly from each other (MWU, *P* = 0.75) and an overall RUD value was thus derived (see Table 3).

### Strawberries: EFSA (2009) crop group “strawberries”

The GD (EFSA 2009) defines strawberries as an independent crop group, requiring its own risk assessment scenarios for birds and mammals (Table 1), but the current default RUD value for strawberries given in EFSA GD (2009) is not actually based on residue samples from strawberries (rather, it is based on residues on grapes). In the underlying database, the residues on strawberries (median 4.5 mg/kg, *n* = 180) differ significantly from those in other berries (median 1.0 mg/kg, *n* = 138) (MWU, *P* = 0.0001). This indicates that strawberries should also be treated as a separate crop in the future.

A risk assessment for frugivorous birds is required in strawberries from flowering through development of fruit to maturity of fruits (BBCH 61–89) in the GD (EFSA 2009). In the data set for strawberries, 138 residue values from growth stages BBCH 73 to 89 were available, covering the entire fruiting period required (principal growth stages 7, development of fruit, to 8, ripening or maturity of fruit and seed). There were no significant differences in RUDs based on samples taken after 1 or 2 applications (MWU, *P* = 0.154) or between S‐EU and C‐EU (MWU, *P* = 0.84; C‐EU data were from Belgium, Germany, northern France, Hungary, the Netherlands, and the United Kingdom; S‐EU data from Spain, southern France, Greece, Italy, and Portugal). Therefore, a single RUD value for strawberries can be recommended (see Table 3). However, the runs test indicated a deviation from a linear model. Significantly fewer runs than expected (46 points above, 93 below the line, and 41 runs) indicate that the relationship between application rate and residue level followed a curve rather than a straight line (RT, *P* = 0.0001).

To check whether the assumption of a linear relationship when using this value in risk assessments would over‐ or underestimate the risk due to exposure, the calculated residues (RUD concept) were compared with the measured residues. An underestimation of the actual residue level should be avoided. An evaluation of the cases in which the predicted values of the linear model are compared with the measured residues showed that in 81% of cases (using a multiple application factor of 1.4 for mean residue data, 2 applications, interval 14 d; see Appendix H in GD EFSA 2009) the predicted values overestimated the measured values. In 19% of cases, the calculated residues underestimated the measured residues with an average of−0.01 mg/kg (3.9%). The assumption of a linear correlation will hence in most cases not underestimate the residues in strawberries and, if it does, only by a small amount. The RUD approach is therefore considered conservative.

## DISCUSSION AND CONCLUSION

The objective of the present project was to reexamine and derive robust initial pesticide residue levels in fruits determined under field conditions as potential diet for birds and mammals according to EFSA (2009) risk assessment principles. Based on a large data set of residue measurements from more than 1000 independent residue trials in total, we were able to obtain relevant data on fruits as diet of birds and mammals from usually ≥100 trials per EFSA (2009) GD‐defined crop group. For the calculation of RUDs, the highest residue values were taken after the last application (not necessarily at the day of treatment). Furthermore, we present specific data on strawberries, which are currently missing in the GD (EFSA 2009) and confirm that the split of fruits from orchards into small and large fruit orchard types (in the GD) is justified from the RUD concept perspective.

The current default RUD values for fruits in the GD are derived from open literature as reviewed by Baril et al. (2005) and are based on a relatively small number of trials (*n* = 9–33, depending on the fruit group). Baril et al. (2005), as the only basis for the current EFSA (2009) RUD default values for fruits, refer to 180 residue values (about half of them from tomatoes) which are divided into 6 different fruit groups, leaving 96 residue values across the remaining groups of fruits covered in this paper. Baril et al. (2005) give no detailed information on methods of application, chemical substance used, or sampling methods. Data from different regions of the world, technologically dependent countries (Europe, North America, Australia, New Zealand), and countries with a greater reliance on human‐powered equipment (Africa, India, Far East, Middle East) were combined. In addition, the data by Baril et al. (2005) cover the time between 1970 and 1999. The number of pesticides and active substances authorized in the EU is constantly changing. Since the 1970s, significant changes in the active substances, formulations, and preparation of spray mixes have occurred. Because the database of Baril et al. (2005) includes studies that are not conducted according to current EU agricultural standards and due to small data sets, the resulting RUD values are of questionable relevance for current European regulatory purposes.

In contrast, the RUD values presented here (see Table 3) are based on 291 studies providing >1000 residue trials. The database available here covers the last 26 y and is therefore more up to date in terms of both the pesticides and the design of the residue study. The latter in particular has undergone significant changes in the course of the last revisions of the GD for risk assessment (Appendix F in EFSA 2009).

All studies used in the present analysis were conducted following regulatory‐relevant study designs and were evaluated by EU member state authorities as being acceptable within the European regulatory processes. Fruit residues sampled on the day of application (or peak residues shortly thereafter) for all required fruiting crop groups are provided (including strawberries). These RUD values are mostly significantly lower compared to the default RUDs (EFSA 2009; see Table 4). However, compared to the current default RUD values, the RUD values presented here are considered to be of higher relevance for European regulatory processes because the underlying residue trials were all conducted in European member states, according to current EU agricultural standards, and the overall data set is much larger.

Regarding the zonal authorization of plant protection products in the EU, various attempts have been made to establish different parameters per regulatory zone for use in the risk assessment (zonal guidance and work sharing documents, e.g., Southern Zone Guidance 2017, Northern Zone Guidance 2018). This may be justified in cases where default or refinement parameters are actually expected to differ between zones (e.g., focal species; see Dietzen et al. 2014). However, as shown in the *Results* section, regarding fruit residues, a subdivision into different zones seems not to be justified or required. In general, there were only small or negligible differences in residue levels between trials conducted in S‐EU and C‐EU. If a statistically significant difference was found (e.g., small fruits from orchards), the absolute difference between mean RUD values was still small. Nevertheless, in these cases, the zonal data set was evaluated separately and the highest value of an identifiable group of fruits is proposed to be taken as default to follow a conservative risk assessment approach (such as Tier 1). Hence, a joint consideration of the data appears more reasonable than to make further distinctions, and we propose to use a single RUD value per fruit group for S‐EU and C‐EU zones in general. This pragmatic approach is in line with the current use of the GD (EFSA 2009) default values and thus ensures easy applicability of the new values in the existing risk assessment procedures.

In terms of fruit type, the data set is not homogeneous in groups combining different fruiting plants such as in orchards, gourds, and berries. Different berries, pome, and stone fruits exhibit a variety of different fruit shapes, sizes, and surfaces. Particularly fruit size, volume–surface area relationship, and details of the skin structure likely influence the residue levels. However, the current division into groups that are jointly regulated seems to generally work in the area of fruit residues because no significant differences were detected among different pome fruits (apple, peach, pear, lemon, mandarin, and orange) or within the group of berries (currants, raspberries, and gooseberries).

In the category of “small fruits from orchards,” different fruits have been combined (apricot, cherry, plum) as per EFSA 2009. Residues from studies with 1 or 2 applications did not differ. However, the data were separated by location for further analysis. In the S‐EU database, most residue values originate from apricots, whereas in the C‐EU the residues were dominated by cherries and plums. Plums exhibited significantly lower residues than did cherries and apricots. Therefore, it could be considered to adapt this in a revised RUD crop grouping. However, in line with current risk assessment practice, we here propose again a single default value for this predefined fruit group (taking the highest value, from cherries in C‐EU, *n* = 44), covering all other small orchard fruits for first tier evaluations (*n* = 192).

For “grapes” and “large fruits from orchards,” it was possible to consider only trials with 1 single application due to the large data set available. For the categories of “other berries,” “gourds,” “small fruits from orchards,” and “strawberries,” studies with more than 1 application were also used to obtain a larger data set. In these cases, values from the day of the last application were taken, representing a conservative estimate of RUD values because RUDs should be highest after the last application. However, as shown in the *Results* section, the number of applications had no significant influence on the residue level in all tested crop groups. This indicates that the selected application intervals of modern plant protection products generally do not lead to a build‐up of residues in fruits but rather maintain an even level to be efficient against the targets.

The GD (EFSA 2009) requires a frugivorous bird scenario (starling) in strawberries, from flowering through development of fruit to maturity of fruits (BBCH 61–89). It is noted that the relevant EFSA scenario for strawberries is not restricted to the actual fruiting growth stages but starts at the beginning of flowering. A separate RUD for strawberry fruits (starting from BBCH 73, Seeds clearly visible on receptacle tissue, to BBCH 89, Second harvest: more fruits coloured) based on corrected crop‐specific data is therefore proposed here to cover this scenario appropriately.

The results of the runs test indicate that the residue data in strawberries deviates from a linear relationship between residue and application rate. Previously published results also indicate that the relationship between application rate and residue concentration might not actually be linear in general (Bennett et al. 1994; Pfleeger et al. 1996). It could indeed be argued that a simple linear relationship between application rate and residues is expected, given the complex relationships between the various parameters that determine residue values (chemical and its metabolism after application, fruit size, surface texture, abiotic factors during application). However, because our aim was to determine new default RUD values for the regulatory processes, this question is beyond the focus of the present work, and we were interested only in whether the assumption of a linear relationship will lead to an underestimation of the true residue values in the model. A more detailed evaluation of the data revealed that the predicted values of the linear model (using the current RUD concept) rather overestimate the measured values. Although our data for strawberries in principle support earlier studies regarding possible nonlinearity (Bennett et al. 1994; Pfleeger et al. 1996), they also suggest that the deviations resulting from the use of a model assuming linearity are likely minimal. For the other fruit groups, linearity was shown.

As shown by Baril et al. (2005), the mean RUD values for fruits were generally much lower (3 to 19 times) than the corresponding values for leaves and shoots. These observations are not unexpected, considering that fruits are most often found within the canopy of fruiting plants. The degree to which the spray is intercepted by leaves may depend to various degrees on the plant morphology, such as size of leaves, canopy density during fruit development, placement and size of the fruits, and the method of spray application.

The study of the fruit residues indicates that fruit residues are based on a complex interaction between fruit characteristics, abiotic factors during application, and chemical properties of the substance. However, the analysis of the linearity in the relationship between application rate and residues confirms that the current RUD concept can calculate expected residues following different application rates with reliable accuracy. Even in cases where the linearity is not given, as in the “strawberry” group, the method will rather overestimate the actual mean residue level. The RUD concept, therefore, can be considered valid and conservative in regard to the reported fruit residues proposed to be used as defaults in first tier risk assessments.

For small orchard and berry fruits, our RUD data sets are of a similar order of magnitude to the data sets of Baril et al. (2005), whereas our RUD data sets for large orchard fruits and pumpkins are very different. The reason for this may be the larger database, which allows for more robust averaging. However, it may also be explained in part by the use of newer products with less active ingredient content, better formulations, and more modern application methods. Unfortunately, it is not possible to make individual comparisons of fruit groups in the 2 databases at present because the original data from Baril et al. (2005) have not been published. In any case, our results seem to support separate RUD categories for strawberry and for grape. The low values indicate that the current extrapolations in the GD (EFSA 2009) are too conservative.

In summary, the present work contributes significantly to bringing more reliability into EU risk assessment procedures for pesticides by assessing residue levels in fruits determined under agricultural field conditions in nearly all fruit‐growing areas of Europe. Based on a large experimental data set of near to or exceeding 100 residue trials per fruit group from studies evaluated in EU member states, considerably lower RUD values compared to the current default EFSA (2009) RUD values were determined. Note that in cases where the value specified in the table was averaged from a smaller subset of data, the specified value still covers a much larger amount of data because it is averaged from the subset with higher residues.

These new RUD values are considered relevant and appropriate for use in wildlife risk assessments of pesticides in Europe. The proposed default values follow the current EFSA (2009) GD crop groups and can replace the current RUD values. However, the large data set also gives the option for further RUD refinement in higher tier assessments regarding fruit‐, growth stage‐, or zone‐specific analysis. Nevertheless, refinement of RUD values per crop will in part lose the benefits of the large data set. Therefore, it is necessary to carefully consider whether the remaining, more crop‐specific, data set is still sufficiently large to justify such refinement.

**Table 4:** Current default RUD values for frugivorous scenarios taken from EFSA (2009) and proposed new default RUD values

Fruit group	Current default RUD values of EFSA (2009) GD (mg/kg)			Proposed new default RUD values (mg/kg)		
	Mean ± SD	90^th^ percentile	Residue values (*n*)	Mean ± SD	90^th^ percentile	Residue values (*n*)
Grapes	8.3 ± 7.25	16.75	9^a^	1.6 ± 1.1	3.3	98
Berries				5.0 ± 3.6^b^	9.2	180
Large fruits from orchards^c^	19.5 ± 16.8	41.1	33	0.9 ± 0.6	1.5	127
Small fruits from orchards	3.3 ± 2.6	6.5	33	2.8 ± 1.3^d^	4.3	44
Gourds	34.3 ± 54.7	61.5	19	0.7 ± 0.7^e^	1.3	209
Strawberries	Not given in EFSA (2009)			1.2 ± 0.7	2.2	138

C‐EU = central European zone; EFSA = European Food Safety Authority; GD = guidance document; RUD = residues per unit dose; S‐EU = southern European zone.

^a^ Residues on grapes and berries (6 trials grapes and 3 trials cane fruits) taken together in the GD (EFSA 2009).

^b^ Currants, raspberries, and gooseberries.

^c^ Apple, peach, pear, lemon, mandarin, and orange.

^d^ RUD value from cherries (C‐EU), covering apricot and plum (C‐EU), and cherry, apricot, and plum (S‐EU); total of 192 trials.

^e^ RUD value from pumpkins, cucumbers, squash, and melons from studies conducted in S‐EU (covering 58 additional RUD values from C‐EU).

## SUPPLEMENTAL DATA

List of fruit residue values used for analysis:

Grapes

Other berries

Gourds

Large fruits from orchards

Small fruits from orchards

Strawberries

## Supporting information

This article contains online‐only Supplemental Data.

Supporting information.

## Data Availability

Supplemental Data, that is, the list of residue on fruits and RUD compilation, may be found in the online version of this article. Requests for data can be sent to the first author.
